# Supplementary material for: The role of organisational- and country-level factors in the volume and public visibility of business and management research
Source: PLoS One. 2024 Jun 11;19(6):e0305162. doi: 10.1371/journal.pone.0305162 (PMC11166347; doi:10.1371/journal.pone.0305162)

**Appendix A**

**A.1. Random Intercepts Model Results for (i) Research Volume and (ii) Public Visibility for (A) High-Income Countries, and (i) Research Volume and (ii) Public Visibility for (B) Low-Income Countries.**

(A) High-Income Countries

(i) Research Volume (ii) Public Visibility


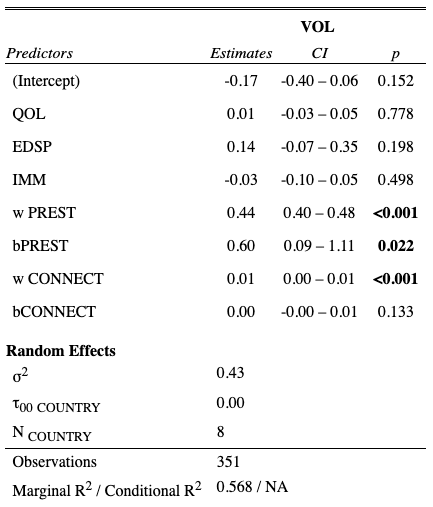

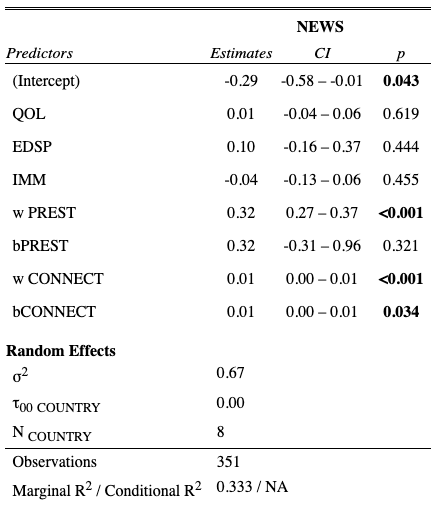


(B) Low-Income Countries

(i) Research Volume (ii) Public Visibility

**
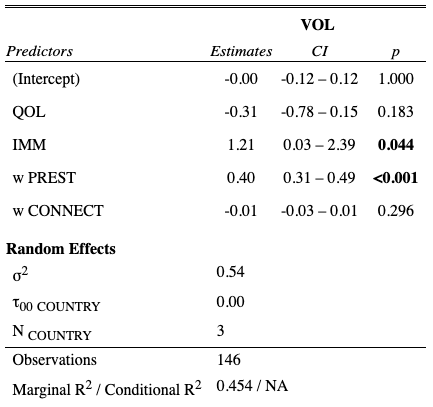

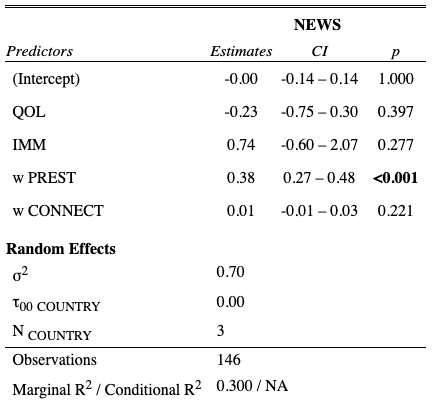
**

**A.2. (Initial) Random Slopes Model Results for (i) Research Volume and (ii) Public Visibility for (A) High-Income Countries, and (i) Research Volume and (ii) Public Visibility for (B) Low-Income Countries.**

(A) High-Income Countries

(i) Research Volume (ii) Public Visibility


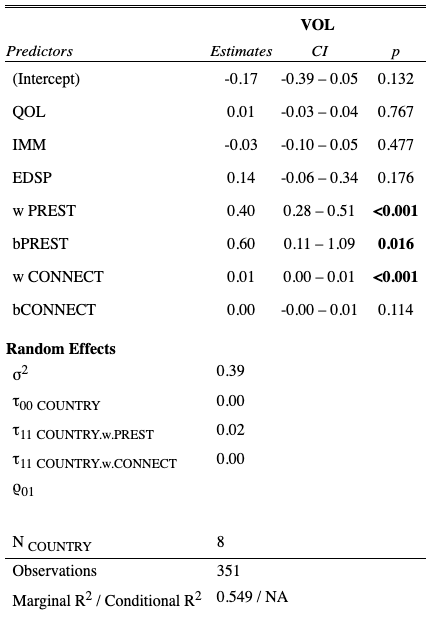

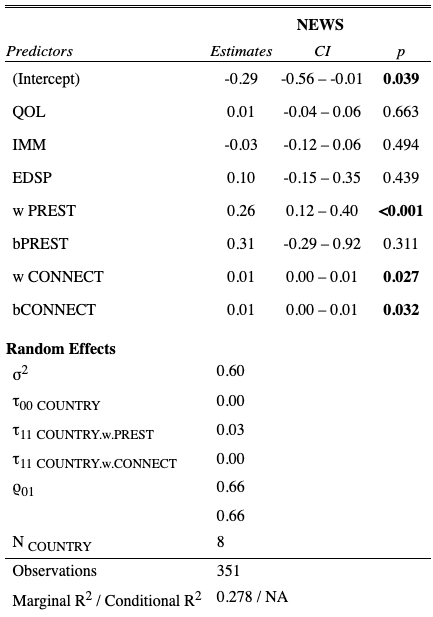


(B) Low-Income Countries

(i) Research Volume (ii) Public Visibility


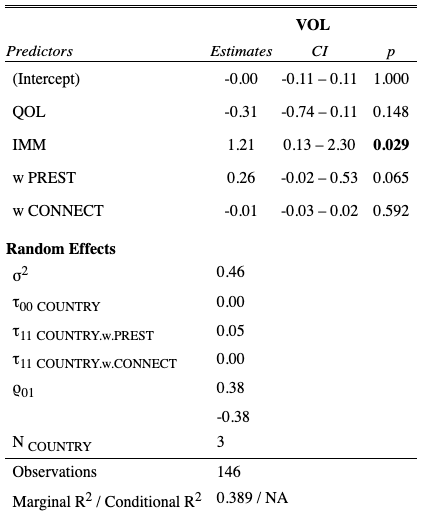

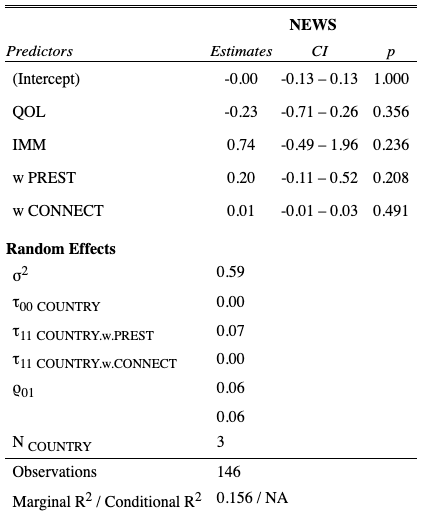


**A.3. Random Effects Significance Tests for (i) Research Volume and (ii) Public Visibility for (A) High-Income Countries, and (i) Research Volume and (ii) Public Visibility for (B) Low-Income Countries.**

(A) High-Income Countries

(i) Research Volume


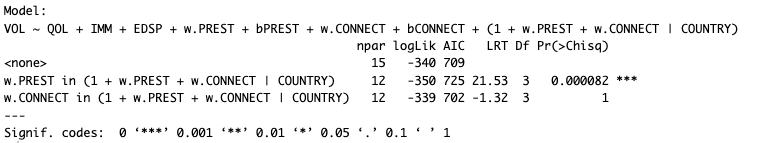


(ii) Public Visibility


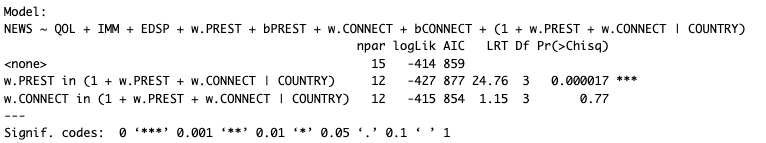


(B) Low-Income Countries

(i) Research Volume


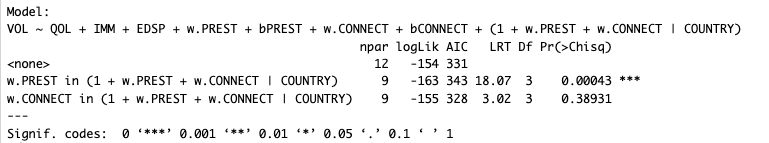


(ii) Public Visibility


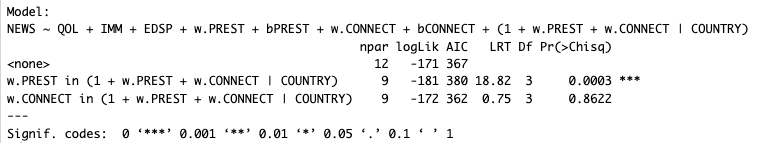


**A.4. Each of the 6 Possible Permutations of Cross-Level Interaction Terms with the Group-Demeaned School Prestige (w.PREST) Variable for the Research Volume Dependent Variable for Both Subsets of Countries:** (i) Quality of Life Index (QOL) x w.PREST, (ii) Percent Recent Immigrants (IMM) x w.PREST, and (iii) Public Tertiary Education Spending (EDSP) x w.PREST for (A) High-Income Countries, and (i) Quality of Life Index (QOL) x w.PREST, (ii) Percent Recent Immigrants (IMM) x w.PREST, and (iii) Public Tertiary Education Spending (EDSP) x w.PREST for (B) Low-Income Countries.

(A) High-Income Countries

(i) QOL x w.PREST (ii) IMM x w.PREST


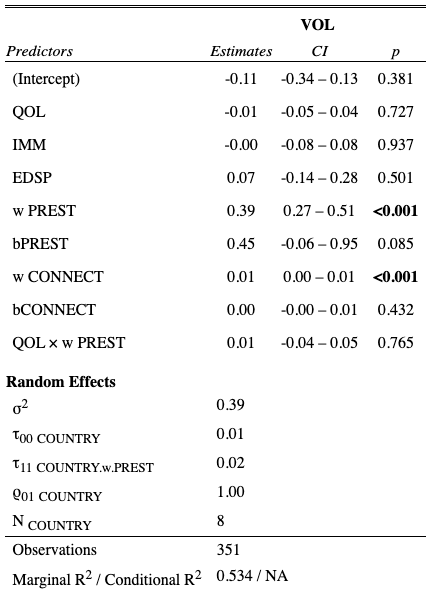

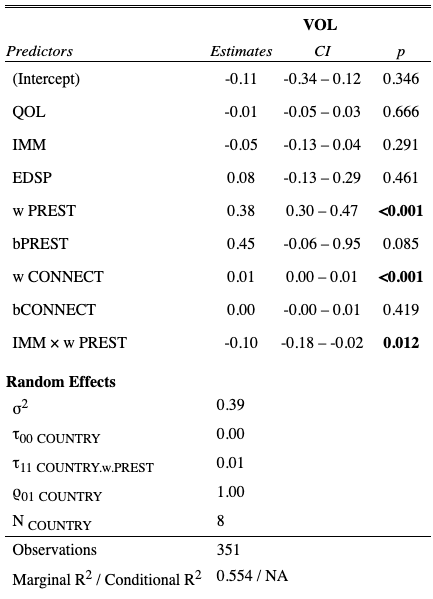


(iii) EDSP x w.PREST


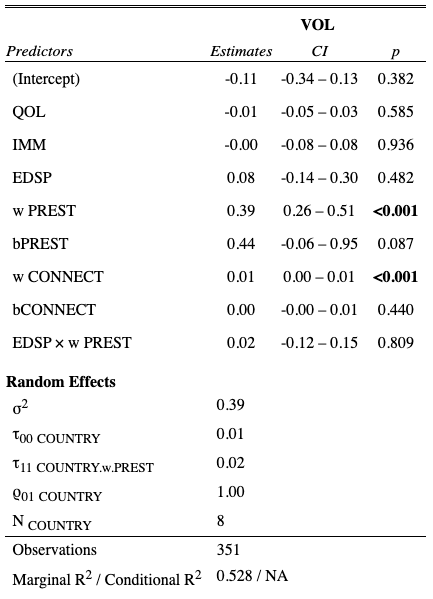


(B) Low-Income Countries

(i) QOL x w.PREST (ii) IMM x w.PREST


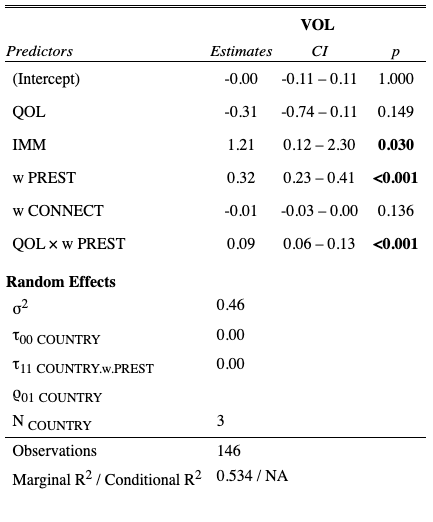

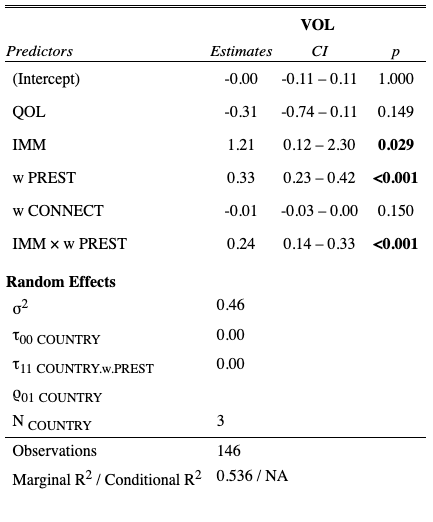


(iii) EDSP x w.PREST


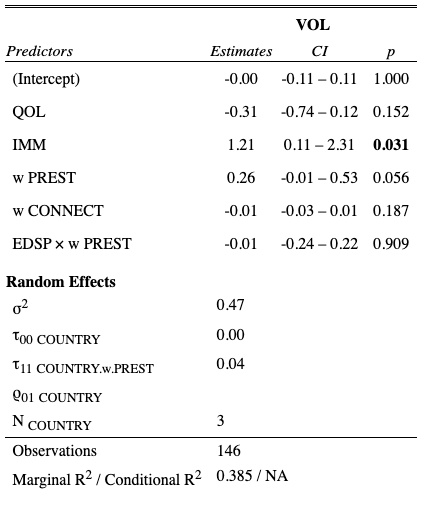


**A.5. Each of the 6 Possible Permutations of Cross-Level Interaction Terms with the Group-Demeaned School Prestige (w.PREST) Variable for the Public Visibility Dependent Variable for Both Subsets of Countries:** (i) Quality of Life Index (QOL) x w.PREST, (ii) Percent Recent Immigrants (IMM) x w.PREST, and (iii) Public Tertiary Education Spending (EDSP) x w.PREST for (A) High-Income Countries, and (i) Quality of Life Index (QOL) x w.PREST, (ii) Percent Recent Immigrants (IMM) x w.PREST, and (iii) Public Tertiary Education Spending (EDSP) x w.PREST for (B) Low-Income Countries.

(A) High-Income Countries

(i) QOL x w.PREST (ii) IMM x w.PREST


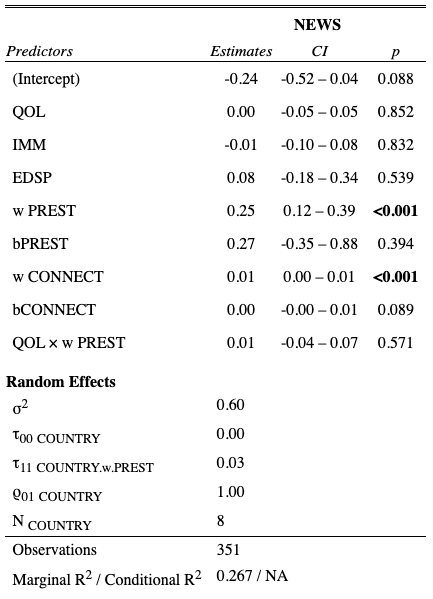

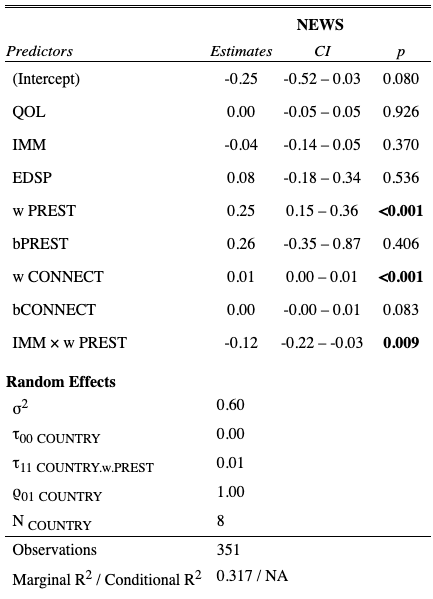


(iii) EDSP x w.PREST


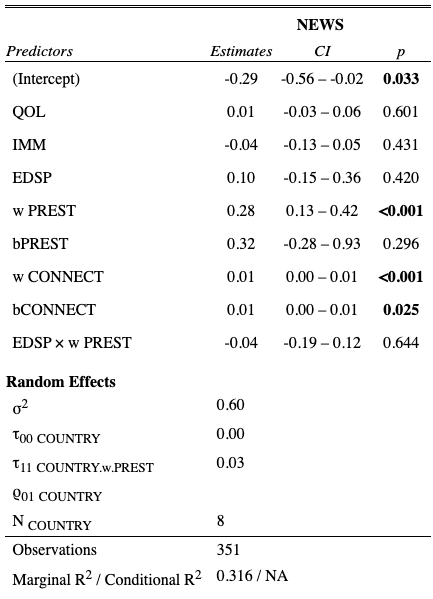


(B) Low-Income Countries

(i) QOL x w.PREST (ii) IMM x w.PREST


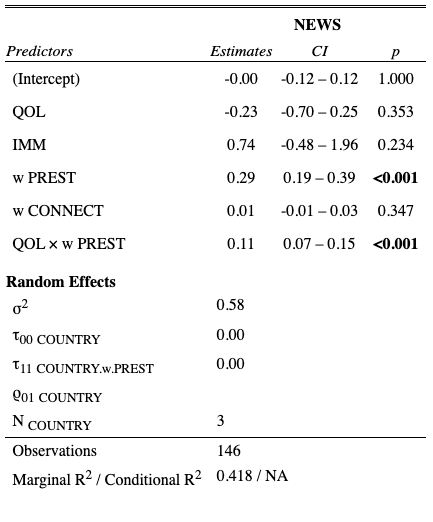

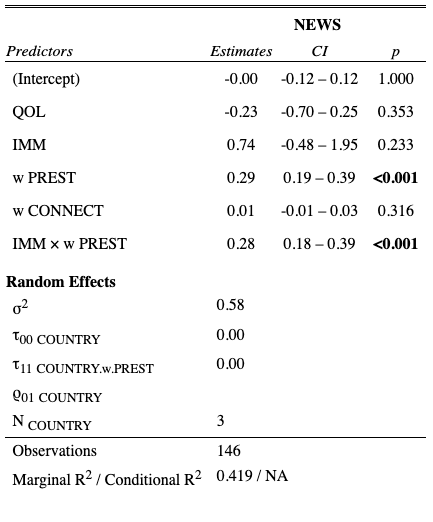


(iii) EDSP x w.PREST


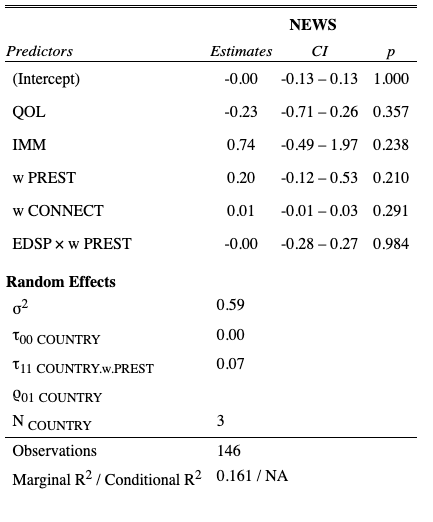


**A.6. Robustness Check Model Results for Each of the 6 Possible Permutations of Cross-Level Interaction Terms with the Group-Demeaned School Prestige (w.PREST) Variable for the Research Volume Dependent Variable for Both Subsets of Countries:** (i) Quality of Life Index (QOL) x w.PREST, (ii) Percent Recent Immigrants (IMM) x w.PREST, and (iii) Public Tertiary Education Spending (EDSP) x w.PREST for (A) High-Income Countries, and (i) Quality of Life Index (QOL) x w.PREST, (ii) Percent Recent Immigrants (IMM) x w.PREST, and (iii) Public Tertiary Education Spending (EDSP) x w.PREST for (B) Low-Income Countries.

(A) High-Income Countries

(i) QOL x w.PREST (ii) IMM x w.PREST


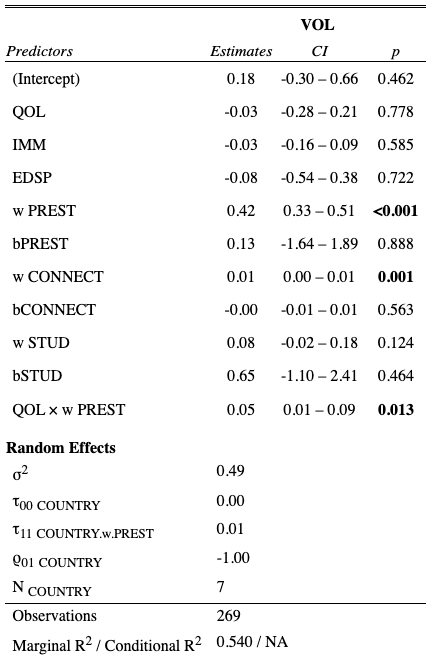

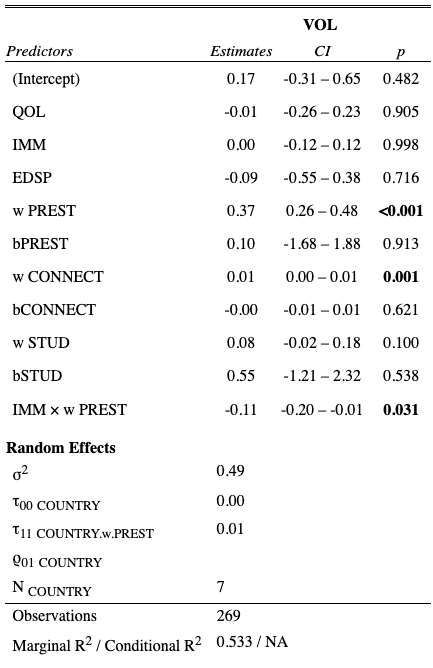


(iii) EDSP x w.PREST


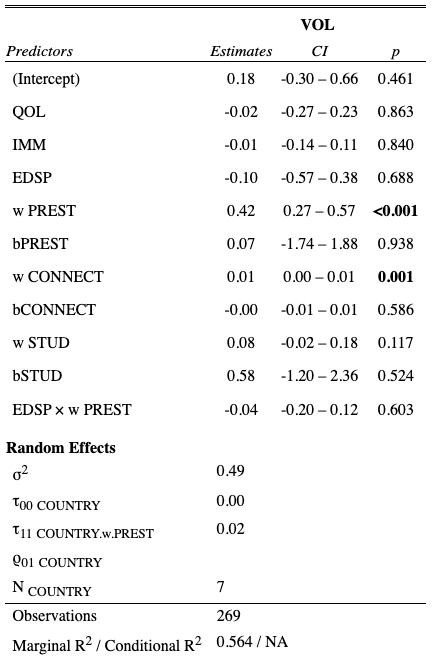


(B) Low-Income Countries

(i) QOL x w.PREST (ii) IMM x w.PREST


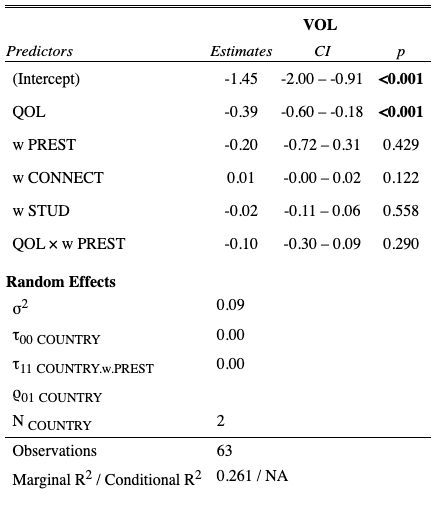

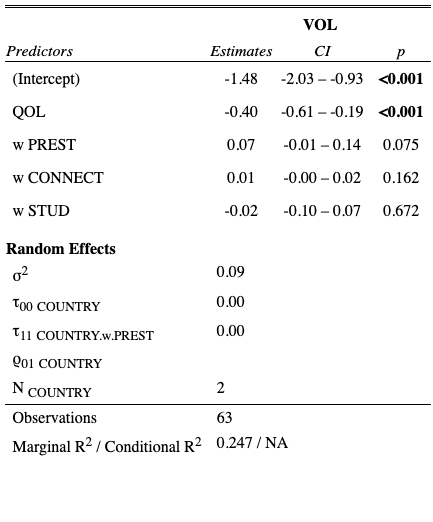


(iii) EDSP x w.PREST


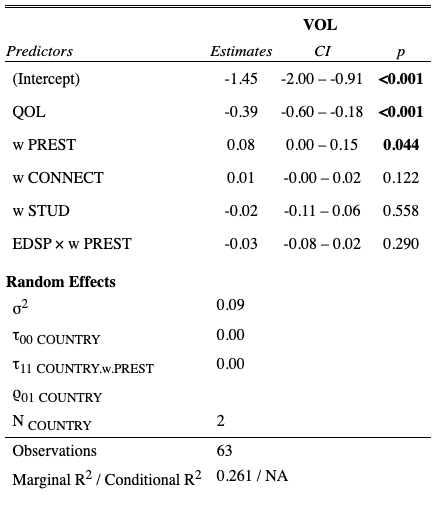


**A.7. Robustness Check Model Results for Each of the 6 Possible Permutations of Cross-Level Interaction Terms with the Group-Demeaned School Prestige (w.PREST) Variable for the Public Visibility Dependent Variable for Both Subsets of Countries:** (i) Quality of Life Index (QOL) x w.PREST, (ii) Percent Recent Immigrants (IMM) x w.PREST, and (iii) Public Tertiary Education Spending (EDSP) x w.PREST for (A) High-Income Countries, and (i) Quality of Life Index (QOL) x w.PREST, (ii) Percent Recent Immigrants (IMM) x w.PREST, and (iii) Public Tertiary Education Spending (EDSP) x w.PREST for (B) Low-Income Countries.

(A) High-Income Countries

(i) QOL x w.PREST (ii) IMM x w.PREST


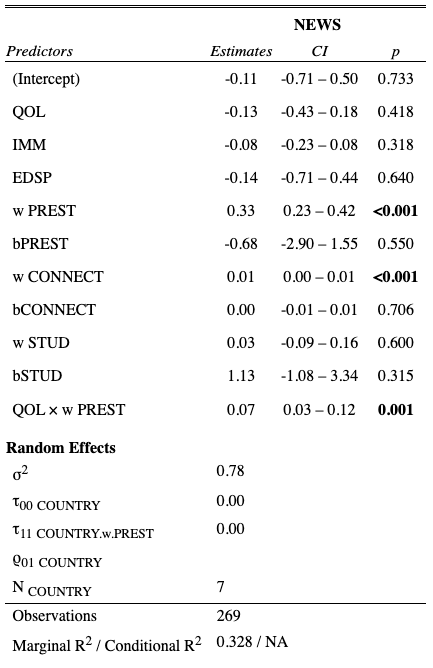

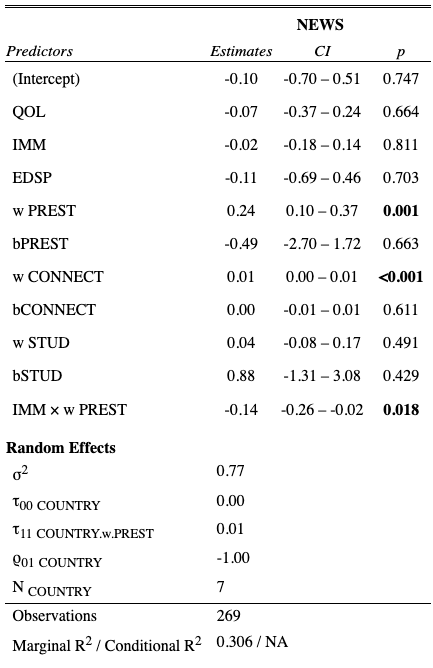


(iii) EDSP x w.PREST


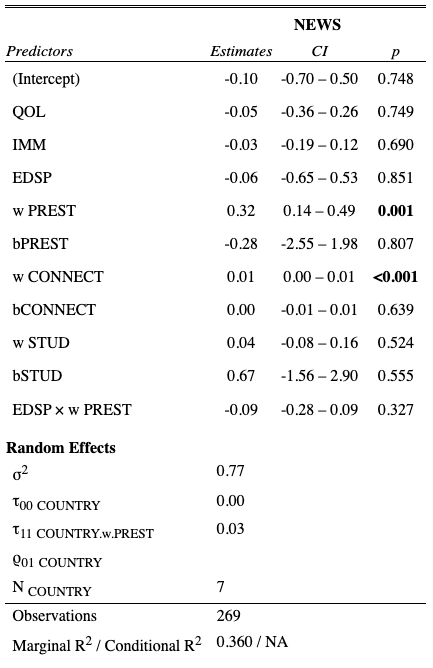


(B) Low-Income Countries

(i) QOL x w.PREST (ii) IMM x w.PREST


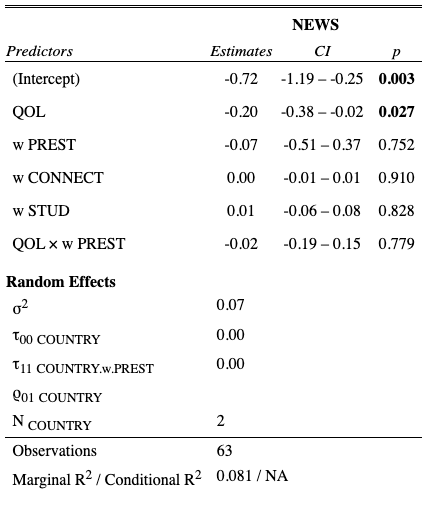

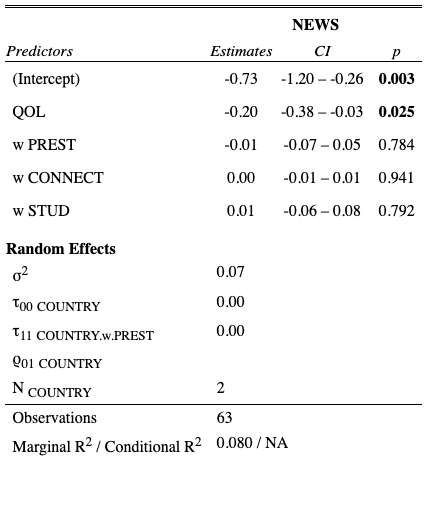


(iii) EDSP x w.PREST


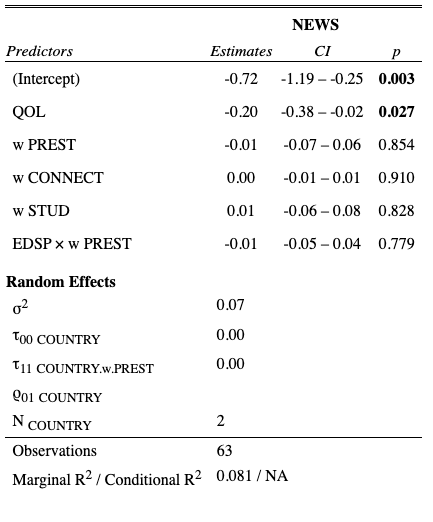

Supplement: S1 File — (DOCX) [file pone.0305162.s001.docx]
