# Supplementary material for: The role of organisational- and country-level factors in the volume and public visibility of business and management research
Source: PLoS One. 2024 Jun 11;19(6):e0305162. doi: 10.1371/journal.pone.0305162 (PMC11166347; doi:10.1371/journal.pone.0305162)
Supplement: Attachment — Submitted filename: Response to Reviewers_R1.docx [file pone.0305162.s002.docx]

**General Response**

We would like to thank the editorial team and the referees for your detailed, helpful and constructive feedback on our paper “Spatial Connectivity, Public Investment, and Immigration: The role of university- and country-level factors in the volume and visibility of business and management research.” We greatly appreciate the time and effort you invested in evaluating our work, and your comments have been immensely helpful in improving the quality of our research.

We have carefully considered each of your comments, discussed them thoroughly, and incorporated them into our latest draft. As you will see in the revised version of the paper, the response to these comments - in particular, the relatively small sample of countries previously examined that both reviewers mentioned - has fundamentally shifted our perspective on the analysis and changed the paper substantially. In the revised manuscript we have included business schools from 4 additional countries in our analysis, which allows us to explicitly leverage the differences in national development level (by splitting the sample into ‘low’- and ‘high’-income countries) to better contextualise and understand the results. This expansion of the analysis (i.e., every result now has a low-income and a high-income version) necessitated some other changes: two new hypotheses have been added to recognise the fact that institutions may respond differently to external factors based on the development level of the country and its overall positioning within the global economic system and highlight the role of prestige in our conceptual thinking. To conserve space and provide a more coherent narrative (in light of the expanded scope of the paper), we have also now removed the analysis of the academic visibility variable.

Interestingly, the new results for ‘high-income’ countries are basically in line with our original results. But now, by splitting the sample - and including two additional low-income countries - we can see that there are substantial differences in the results for ‘low-income’ countries. Overall, we think that this new approach significantly improves the paper and enhances what we can take away from the analysis.

In addition, we have contextualised the paper in terms of the new geopolitics of higher education, further clarified some of the indicators used in the analysis, made changes to the figures and tables, and edited the title and abstract to reflect the results of the new analysis.

We attach the most recent draft of our paper (with changes tracked) and a detailed response to each of the editor's and reviewers' specific comments and concerns to demonstrate how we have incorporated their feedback.

Once again, thank you for your valuable contributions to our research.

**Editor Comments**

Thank you for submitting your manuscript to PLOS ONE. After careful consideration, we feel that it has merit but does not fully meet PLOS ONE’s publication criteria as it currently stands. Therefore, we invite you to submit a revised version of the manuscript that addresses the points raised during the review process.

This is a timely and fine research piece, but it needs a major revision from several angles. Both reviewers provided a very detailed critical feedback with important comments that need to be addressed properly. As Rev2 points out, both the Introduction and the methodological parts require refinement. Also, for the broader geopolitical picture (esp. connected with HE), some fresh pieces of scientific papers should be looked at, referred to critically and embedded in the body of the analysis. See these, for instance:

<https://www.tandfonline.com/doi/abs/10.1080/14767724.2023.2166465>

<https://link.springer.com/book/10.1007/978-3-030-94415-5>

<https://ecpr.eu/Events/Event/PanelDetails/9963>

<https://www.tandfonline.com/doi/abs/10.1080/03098265.2022.2045572>

***Response:*** *Thanks for these comments, and specifically the suggestion to incorporate the literature on the new geopolitics of higher education. We agree that it makes sense as a lens through which to view our analysis of country-level factors and research performance. We’ve now added a paragraph referencing the role of geopolitics in higher education policy in the introduction on p. 2 of the revised manuscript with several of the citations you mentioned here.*

**Reviewer Comments**

*Reviewer #1*

In general, it can be ascertained that the study deals with a very relevant topic, at an appropriate professional level, primarily based on quantitative research methods. All in all, and taking into account the comments below, the study is suitable for publication with minor revisions and modifications.

The introduction outlines the scope of the study and also points out its novelty and some of its practical and theoretical aspects, however, this part gives the impression of a more detailed and extensive abstract, or a summary of the research results (than an introduction), as it describes the aims of the study, addresses its main methodological issues, and presents the conclusions (at the same time, it contains fewer literature antecedents and references), which isn’t an issue if the editorial board accepts this approach.

In connection with this chapter, the reviewer recommends that the authors explain in more detail who has dealt with this topic in the past, with what methods, and what the most important findings of previous research related to the topic. It would be appropriate to close the chapter by presenting the objectives of the research.

The study partially fulfils the above „task” in the chapter titled "Spatial distribution of research performance", which essentially details the theoretical framework of the research topic, as well as clarifying the objectives and presenting and formulating the hypotheses. At the same time, the range of sources could be expanded here, especially in connection with findings related to innovation (for example Asheim, B. T., Coenen, L. (2006): Contextualising regional innovation systems in a globalising learning economy: On knowledge bases and institutional frameworks. The Journal of Technology Transfer, 1., 163-173. https://doi.org/10.1007/s10961-005-5028-0; Bathelt, H., Malmberg, A., Maskell, P. (2004): Clusters and knowledge: Local buzz, global pipelines and the process of knowledge creation. Progress in Human Geography, 1., 31-56. https://doi.org/ 10.1191/0309132504ph469oa; Godin, B., Lane, J. P. (2013): Pushes and pulls: Hi(S)tory of the demand pull model of innovation. Science, Technology & Human Values, 5., 621-654. https://doi.org/10.1177/0162243912473163; Oláh, D. ; Alpek, B. L. (2021): The theoretical model of spatial production for innovation. Journal of Innovation and Entrepreneurship 10 (1) https://doi.org/10.1186/s13731-021-00182-4.

***Response:*** *Thanks for these comments. Regarding the organisation of the paper and the structure of the introduction, we typically found it useful to use the introduction to provide 1) a broad conceptual/practical frame for the paper, then 2) a direct statement of its research questions, and finally 3) a synthesis of the results and key findings. We then use later sections (the ‘literature review,’ ‘conceptual framework,’ or, here, ‘spatial distribution of research performance’) to explore the existing literature on a given topic in more detail.*

*Given that overall structure, we’re very happy to include the relevant citations that you mention in the sub-section ‘University location as a predictor of research performance.’ These are very useful papers that are now included in our theoretical discussion (however brief) of agglomeration and innovation on p. 6 of the revised manuscript.*

The finding regarding the relationship between quality of life and academic performance is one of the surprising results of the research. In this context, it would be worthwhile to present the authors' approach to the quality of life when describing the hypotheses. Later, the study provides an answer to this in the methodological part, but since many factors not included in the present study, but related to the quality of life, are also mentioned in the description of the hypothesis, it would be worthwhile to emphasize the approach used in the study.

***Response:*** *Good point - it certainly makes sense to define how we’re measuring quality of life up front for the reader. A brief description of the quality of life measure used in the paper has been added to the introduction of the revised manuscript on p. 4.*

The presentation of the methods is precise and detailed, and the methodological approach is appropriate. However, the section can be further developed along the lines of the following findings. On the one hand, the steps and methodological solutions included in the source cited by the authors should be presented in more detail. In connection with the selection of the sample, it should be made clearer why these countries were chosen and why the authors decided not to investigate (at least) the EU countries, either by expanding the range of journals or countries. Why is it missing from the analysis, for example, France or Germany? It is conspicuous to the reader that the weight of the United Kingdom is strikingly high in terms of the number of institutions among the selected countries (40%), and that all the selected countries are very close to each other in terms of development and quality of life (nearly the same HDI, etc.). The question arises whether, for example in terms of the quality of life: To what extent would the results of the models differ from the current ones if countries with a lower quality of life were also included in the sample?

***Response:*** *Thanks for this important comment - we agree that the sample of countries included in the original draft was somewhat limited in scope, which may have hindered our ability to generalise the results. Our revised manuscript makes a substantial improvement to address this shortcoming. Following your advice, we added to the sample two countries with generally lower quality of life (Poland and Greece). Following Reviewer 2’s guidance, we added to the sample two more countries (Belgium and the Netherlands). This increased the sample size by 57% at the country-level (from y to 11 countries) and by 38% at organisational-level (from 360 to 497 organisations). Adding data for four countries in the revised manuscript enabled us to expand the range of observed income levels/quality of life.*

*As you will see in the revised version of the paper, the inclusion of these countries fundamentally shifted our perspective on the analysis and changed the paper substantially. To explicitly leverage the differences in development level between these countries, we have now decided to split the data into two subsets and compare the analysis: business schools in high- and low-income countries (those with GDP per capita above and below the EU average in 2018, respectively). This expansion of the analysis (i.e., every result now has a low-income and a high-income version) necessitated some other changes: two new hypotheses have been added to recognise the fact that institutions may respond differently to external factors based on the development level of the country and its overall positioning within the global economic system and highlight the role of prestige in our conceptual thinking. To conserve space and provide a more coherent narrative (in light of the expanded scope of the paper), we have also now removed the analysis of the academic visibility variable. In general it behaves less consistently (in terms of its relationship to the predictors) than the other two dependent variables, which we think may be due to its construction as a rate (visibility-adjusted volume divided by volume) compared to the other two, which are counts. However, we are happy to include those results if desired. Finally, we also had to create some new composite measures (e.g., of school prestige) to simplify the number of comparisons being made in the models.*

*Interestingly, the new results for ‘high-income’ countries are basically in line with our original results. But now, by splitting the sample - and including two additional low-income countries - we can see that there are substantial differences in the results for ‘low-income’ countries.*

*As before, for high-income countries, immigration influences research volume and public visibility primarily through its interaction with prestige: less prestigious business schools appear to benefit the most from higher rates of recent international immigration. Spatial connectivity to other universities and major transportation connections is also a significant driver of volume and visibility in these countries. However, in low-income countries spatial connectivity does not appear to play a significant role in research outcomes, and the relationship with immigration works in a different way: in these countries, more prestigious schools benefit the most from higher rates of recent international immigration (although all schools benefit to some extent). In addition, in low-income countries, a composite measure of a country’s quality of life is negatively related to research outcomes through its interaction with school prestige. Thus in low-income countries with lower quality of life, prestige isn’t a significant differentiator for research productivity. However, in low-income countries with higher quality of life, researchers at less prestigious schools produce much less than those at more prestigious schools. In this way, we have found that the counterintuitive result in the previous version of the manuscript related to quality of life is only significant in the low-income subset of countries, and only there through its interaction with prestige. This provides more nuanced insight into the way that quality of life might be affecting research performance.*

*Overall, we think that this new approach significantly improves the paper and enhances what we can take away from the analysis. We are grateful to the reviewer for the advice which proved so valuable for the development of our manuscript.*

The description of the indicators and calculation procedures is precise and detailed. It is particularly useful from the point of view of the results that the authors describe the range of selected indicators and sub-indicators in a clear form, and they are also reviewed in a summary table. Either as a future research direction or in the context of this research, it would be worthwhile to also take into account the proximity and number of other institutions directly or indirectly related to research performance (for example research centres, innovation centres, the size of the number of enterprises operating in the high-tech sector), and expand the range of indicators in connection with the measurement of quality of life. Regarding the robustness check, the authors mention that “Finally, robustness checks are made for each of the final models with the number of students per school…”. How much it would have been more suitable, and how much it would have led to other results using the number of lecturers/researchers instead of the number of students?

***Response:*** *Thanks for these comments - we agree that these would provide for useful robustness checks. However, the uniqueness of our dataset, which is manually constructed from multiple archival sources, comes at the cost of limited access to some additional variables, which could potentially serve as robustness checks. For example, while the number of students could be obtained from the (proprietary) AACSB database and from some national statistics agencies, we could not find data sources that would provide consistent and reliable information on the number of faculty employed in business schools. Similarly, collecting information on the number of research centres would require an entirely new substantial data collection effort that we feel is beyond the scope of this paper.*

*In terms of changes to the results, we would not expect that including the number of faculty as a control would significantly change the results we show here - compared to the robustness check using students - as the number of faculty and students are likely to be highly-correlated across business schools. However, it would be useful to analyse more concretely in future extensions of this research. We have added a sentence on p. 33 of the revised manuscript that specifically identifies these items as areas for future extensions of this research.*

The presentation of the results is accurate and clear. A special advantage is that the study emphasizes the limitations of the research in detail and clearly, thereby allowing the reader to interpret the study's results in an appropriate context. In addition to all of this, it would be worthwhile to draw attention to the content of the indicators even more prominently in the introductory and summary sections (as the authors do with the hypotheses). In addition, it could be emphasized even more that the investigation focuses on a special scientific field and region.

***Response:*** *Yes, this is a good point. We have made a few efforts to further clarify the specific discipline, region, and indicators used in the paper. First, we replaced the general use of the term ‘universities’ with ‘business schools’ in most cases so that the reader is reminded that this analysis deals with a specific discipline. On p. 34 of the revised manuscript we have added a sentence clarifying the special focus of the paper: “Despite these limitations – and keeping in mind that the results pertain specifically to business and management research in 11 European countries using the available data collected – we think that this analysis can have several important implications for local and national policy.” We have also included brief descriptions of the composite indicators when discussing the results in the introduction and conclusion.*

In my opinion, it could be an interesting and useful research direction for the extension of the research to other scientific fields and regions.

***Response:*** *Thank you for this suggestion. We have added a mention of future extensions to “other scientific disciplines” on p. 33 of the revised manuscript.*

About figures and tables, it would be recommended to insert a summary line in Table 1, as well as display the share of each country within the sample. The texts of the figures are very small and therefore difficult to read properly. Similarly, the maps and texts in Figure 4 are difficult to see. All in all, the tables and figures in the study are informative and easy to interpret.

***Response:*** *We have updated Table 1 to include each country’s share of institutions and the total number of institutions, as well as information on the GDP per capita and whether it is a ‘high’- or ‘low’-income country in respect to our updated analysis. We have also tried to increase the size of the text in the figures as much as possible. This is a very important comment, and, given the limited space available in a journal publication, it is always difficult to effectively convey as much information as possible (e.g., in a map) while still having it be readable in a static format. If you still feel the text is too small, we can make more difficult choices about completely reformatting some figures and/or putting them in the appendix (or, e.g., online-only).*

In general, the study deals with an interesting and useful topic, based on a suitable methodological base. Although it would be interesting and worthwhile to expand the horizon of the research in several aspects with minor modifications, the study is suitable for publication even in its current form.

***Response:*** *Thank you, we appreciate your support and are thankful for your guidance.*

*Reviewer #2*

Spatial Connectivity, Public Investment, and Immigration: The role of university- and country-level factors in the volume and visibility of business and management research

This paper focuses on the examination of the role of universities, research centres, organizations that play role in helping scholars to achieve better results. Educational and research institutions can be examined from different point of views like spatial constellation, their role at different levels, moreover being an engine of regional and local collaborations, innovations and initiatives, an outstanding potential employer, probably the largest one. To me authors undertake too much factors in the introduction. As the title also indicates, too long, too complicated, readers including me do not understand the point of their paper clearly. My suggestion would be for the authors to clarify the purpose and focus of the study by attempting to define a narrower scope.

***Response:*** *We appreciate your concern about the paper’s focus in the introduction and title - we are certainly testing a number of different relationships here, and we can see how it might be a bit overwhelming to the reader. To improve readability, we followed your advice and shortened the title of our paper, which now reads “The role of organisational- and country-level factors in the volume and public visibility of business and management research.” We also added three sentences to the first paragraph of introduction, which seek to focus the reader’s mind on the main broad questions we address in our paper. The purpose of the paper is stated on p. 3, “to empirically analyse the comparative importance of the features of academic institutions themselves and their national societal contexts in predicting research outcomes.”*

*That said, the purpose (and, we think, strength) of the paper is that it examines the impact of a variety of factors, at both the organisational- and country-level, on research performance. Our results are necessarily somewhat nuanced and context-dependent. Given that nuance, our inclination is to not over-generalise in the abstract, introduction, or conclusion, so that the reader understands the specific nature of the empirical results – rather than assuming the paper examines one kind of relationship and being disappointed when they find out it doesn’t.*

*We hope that the changes we made have allowed us to reach a balance between complexity and clarity. We are happy to follow your further guidance on how to improve this balance.*

In the first sentence on the 3^rd^ page authors refer to the literature that explores a broad range of organizational factors, but without citation. They should put the most relevant citations/references over there, what sort of literature analyze the aforementioned factors actually. Later on they could refer back to these review even picking the current citation or statement they have mentioned.

***Response:*** *Many thanks for this suggestion, which we are happy to follow. We now cite a recent literature review of organisational factors influencing research productivity (Ryazanova & Jaskiene, 2022) on p. 1 and p. 5 of the revised manuscript. We also cite a number of other relevant papers in the section leading to H1 on p. 5.*

In regard to H1 assumption I am afraid I need to say this is an axiom, this is not a real question/hypothesis.

***Response:*** *We appreciate that the extent to which proximity to transportation resources and other organisations increases knowledge spillovers, innovation, and research output (i.e., agglomeration economies) is one of the most studied questions in regional science, spatial scientometrics, and economic geography. The hypothesis you are referring to (H2 in the revised manuscript) reflects our assumption, based on this literature, that proximity to other business schools, air, and rail service will relate to higher levels of research output. However, we believe that this is not axiomatic, because every location varies in respect to its spatial proximity to the rest of the world (i.e., some places are more or less well-connected), and it is not immediately clear that the relationships found for other types of organisations would also apply to business schools (although we hypothesise that they would).*

*To make our hypothesis sound less like an axiom, we have now revised H2 to say that in this paper we hypothesise that “Greater spatial connectivity of the business school location is positively related to research performance (volume of publication and public visibility).”*

In the case of H2, I think this assumption must be better, because positive implications of funding, subsidies, financial support could already be measured. However there are more than 25,000 universities across the world. There is no one research centre that could develop models for analysing all of them. Conditions, circumstances, endowments are so deeply different, even if you dealt with the American, or British, or both system, but universities in general? If you might fix your intro and hypothesis, make them much more precise - by adding clarifying thoughts - your statements would be also more valid.

***Response:*** *Many thanks for this comment. We agree that there is substantial diversity in the university sector. To a large extent, our study is motivated by the objective to make sense of this diversity, or, at least, some of its major sources. Both our theoretical model (the choice of variables) and our methodology (multilevel design) are designed with the purpose to predict outcomes in a diverse sample of institutions. Our sample is contained within one (albeit broad and internally diverse) discipline of business/management, which we state clearly in our introduction on p. 3. To further underscore this point, we have also now made sure that we talk about business schools (rather than universities) in our hypotheses and throughout the text. Given that there are universities that produce research in business/management without having a business school as a unit, this is a simplification, but we feel that improved readability justifies it on this particular occasion, because “business schools” have become a shorthand for any department teaching and researching business and management. We hope it resolves your concern about over-generalisation.*

About H3. Authors use the concept "immigration", but they should clarify again that migration of who? What kind of people, from which areas? Scientists? Or physical labor force? Or students? Or qualified labor force like doctors, engineers, researchers?

***Response:*** *Many thanks for motivating us to improve the clarity of this construct for a reader, which we do in the revised manuscript.* *The immigration variable comes directly from Eurostat and is defined on p. 15 of our manuscript: it is defined as the number of recent immigrants aged 15-64 from any foreign country as a percent of the receiving country’s total population. To further clarify, we have now included this full definition of the variable when we mention it in the introduction (p. 4) and conclusion (p. 30).*

In Methodology authors already refer to the countries they have picked for having a picture on universities' role, but these countries are European countries, more precisely the Scandinavian countries, the British islands and Spain as a cuckoo egg. I am afraid I do not understand why authors compare these state's universities, why they have not chosen the Netherlands or Belgium, or even Germany as a subject for their research.

***Response:*** *Thank you for this important comment, which shaped the direction of our revision. Following your advice, we added both Belgium and the Netherlands to our sample. We also added two European countries with lower income and lower quality of life (Poland and Greece), as advised by Reviewer 1. This increased the sample size by 57% at country level (from seven to 11 countries) and by 38% at organisational level (from 360 to 497 organisations). Adding data for four countries in the revised manuscript enabled us to expand the range of observed income levels/quality of life, as well as increasing the internal diversity of the sample.*

*As you will see in the revised version of the paper, the inclusion of four new countries fundamentally shifted our perspective on the analysis and changed the paper substantially. To explicitly leverage the differences in development level between these countries, we have now decided to split the data into two subsets and compare the analysis: business schools in high- and low-income countries (those with GDP per capita above and below the EU average in 2018, respectively). This expansion of the analysis (i.e., every result now has a low-income and a high-income version) necessitated some other changes: two new hypotheses have been added to recognise the fact that institutions may respond differently to external factors based on the development level of the country and its overall positioning within the global economic system and highlight the role of prestige in our conceptual thinking. To conserve space and provide a more coherent narrative (in light of the expanded scope of the paper), we have also now removed the analysis of the academic visibility variable. In general it behaves less consistently (in terms of its relationship to the predictors) than the other two dependent variables, which we think may be due to its construction as a rate (visibility-adjusted volume divided by volume) compared to the other two, which are counts. However, we are happy to include those results if desired. Finally, we also had to create some new composite measures (e.g., of school prestige) to simplify the number of comparisons being made in the models.*

*Interestingly, the new results for ‘high-income’ countries are basically in line with our original results. But now, by splitting the sample - and including two additional low-income countries - we can see that there are substantial differences in the results for ‘low-income’ countries.*

*As before, for high-income countries, immigration influences research volume and public visibility primarily through its interaction with prestige: less prestigious business schools appear to benefit the most from higher rates of recent international immigration. Spatial connectivity to other universities and major transportation connections is also a significant driver of volume and visibility in these countries. However, in low-income countries spatial connectivity does not appear to play a significant role in research outcomes, and the relationship with immigration works in a different way: in these countries, more prestigious schools benefit the most from higher rates of recent international immigration (although all schools benefit to some extent). In addition, in low-income countries, a composite measure of a country’s quality of life is negatively related to research outcomes through its interaction with school prestige. Thus in low-income countries with lower quality of life, prestige isn’t a significant differentiator for research productivity. However, in low-income countries with higher quality of life, researchers at less prestigious schools produce much less than those at more prestigious schools. In this way, we have found that the counterintuitive result in the previous version of the manuscript related to quality of life is only significant in the low-income subset of countries, and only there through its interaction with prestige. This provides more nuanced insight into the way that quality of life might be affecting research performance.*

*We think that this new approach significantly improves the paper and enhances what we can take away from the analysis. We are sincerely grateful for your advice which proved so valuable for the development of our manuscript.*

If authors clarify all problematic parts (introduction and hypotheses), their paper's quality will be much higher, much greater therefore I recommend them to fix these shortcomings and defaults.

***Response:*** *We hope that the substantial changes in the manuscript, guided by your advice and the advice of Reviewer 1, have resolved your concerns. We are happy to continue improving our manuscript if needed.*
